# CD38 prognostic role in chronic lymphocytic leukemia patients treated with standard chemotherapy or targeted agents: a monocentric real-life experience

**DOI:** 10.3389/fonc.2025.1569707

**Published:** 2025-05-08

**Authors:** Laura Mettivier, Danilo De Novellis, Anna Maria Della Corte, Bianca Serio, Luca Pezzullo, Roberto Guariglia, Idalucia Ferrara, Raffaele Fontana, Maria Carmen Martorelli, Serena Luponio, Maria Teresa Buonanno, Rossella Marcucci, Valentina Giudice, Carmine Selleri

**Affiliations:** ^1^ Hematology and Transplant Center, University Hospital “San Giovanni di Dio e Ruggi d’Aragona”, Salerno, Italy; ^2^ Department of Medicine, Surgery, and Dentistry, University of Salerno, Baronissi, Italy

**Keywords:** chronic lymphocytic leukemia, chemotherapy, targeted therapy, real-life, personalized medicine, CD38

## Abstract

**Introduction:**

Therapeutic strategies for patients affected by Chronic Lymphocytic Leukemia (CLL) have undergone significant changes over the last decade, shifting from chemoimmunotherapy to targeted therapy.

**Methods:**

This retrospective, single-center, real-word study aims to identify candidate prognostic markers in 230 consecutive CLL patients treated with standard chemoimmunotherapies or targeted agents from July 2011 to June 2023.

**Results:**

Patients receiving targeted therapy were more likely to have mutated *IGHV*, while those with a CD38^+^CD49d^+^ CLL immunophenotype showed an increased risk of refractoriness and disease recurrence, as demonstrated by multivariate analysis. Conversely, CLL patients with a CD38^-^CD49d^-^ phenotype received great benefits when treated with targeted agents, whereas advanced age was a negative risk factor for patients treated with standard chemotherapy.

**Conclusions:**

In conclusion, CD38 expression emerges as a key prognostic marker in CLL, reinforcing the need to integrate clinical, biological, phenotypic, and molecular factors into treatment decision-making and both standard chemotherapy and targeted regimens remain effective in real-life settings.

## 1 Introduction

Chronic lymphocytic leukemia (CLL) and small lymphocytic lymphoma (SLL) are the most common B cell lymphoproliferative disorders originating from mature CD5^+^CD19^+^CD23^+^CD20^+^ B lymphocytes and are characterized by accumulation of neoplastic cells in peripheral blood, lymph nodes, spleen, and bone marrow (1, 2). The majority of patients can benefit from a “watch & wait” approach, while those requiring therapeutic treatments can achieve a 5-year overall survival (OS) rate of up to 87.2%, largely due to the introduction of several novel targeted therapies, such as Bruton’s Tyrosine Kinase inhibitors (BTKi), B-Cell Lymphoma-2 inhibitors (BCL2i), and Phosphatidylinositol 3-kinase inhibitor (PI3Ki), often in combination with anti-CD20 monoclonal antibodies as first and subsequent lines of therapy, resulting in significant improvements of clinical outcomes (3, 4). Therefore, standard chemotherapy has been progressively replaced with targeted agents (3). Despite these therapeutic advancements, CLL/SLL remains an uncurable disease, as most patients eventually experience disease relapse, except for those who benefit from curative allogenic stem cell transplantation (5, 6).

Because of their remitting-relapsing clinical history, CLL/SLL patients require multiple lines of therapies with different mechanisms of action, influencing treatment responses and long-term outcomes. Current treatment algorithms consider *TP53* and *IGHV* mutational status and patient’s fitness for choosing the best personalized therapeutic approach (7); however, CLL is a heterogeneous group of B cell lymphoma, with various clinical, phenotypic, cytogenetics, and molecular characteristics, affecting clinical outcomes (8). Therefore, a better risk stratification and identification of markers of responsiveness to BTKi and BCL2i are two important unmet clinical needs to optimize personalized treatment approach in CLL/SLL patients. In this scenario, real-world evidence plays a crucial role in assessing the best treatment sequencing. Additionally, several clinical and biological criteria, such as age, fitness, comorbidities, cytogenetic and molecular abnormalities, and disease aggressivity have been identified over time to select the most appropriate therapeutic option (9).

In this single-center retrospective study, we described CD38 prognostic role in CLL patients treated with standard chemotherapies or anti-CLL agents, with various therapeutic sequencing approaches in a real-life setting over a ten-year follow-up period at Hematology and Transplant Center, University Hospital “San Giovanni di Dio e Ruggi D’Aragona”, Salerno, Italy. The impact of CD38 expression and other known prognostic markers on treatment choice and clinical outcomes was explored, including time to next treatment (TTNT), time to next treatment failure or death (TTNF), progression-free survival (PFS), OS, and hematological responses, with a focus on differences in patients treated with targeted therapy, standard chemoimmunotherapy, or on observation were investigated.

## 2 Materials and methods

### 2.1 Patients

A total of 230 consecutive CLL patients were included in this single-center retrospective real-life study, performed at Hematology and Transplant Center, University Hospital “San Giovanni di Dio e Ruggi D’Aragona”, Salerno, Italy, from July 2011 to June 2023. Patients received a diagnosis of CLL/SLL according to international guidelines and were on observation or treatment based on International Workshop on CLL (iwCLL) guidelines (9). Treatment choice was based on medical decision, patient’s status, available clinical, molecular, and phenotypic characteristics, and patient’s preference. Hematological response was assessed at the end of each treatment according to iwCLL criteria, and patients were monitored every 3–6 months by flow cytometry immunophenotyping. Clinical characteristics of the entire cohort are displayed in **Table 1**. Treatment duration was as per international guidelines for fixed schemes (e.g., six cycles of fludarabine, cyclophosphamide, and rituximab), while for alkylating agents or BTKi, treatment was administered until disease progression or severe toxicities.

**Table 1:** Clinical characteristics at diagnosis.

Characteristics	N = 230
Median age, years (range)	70 (39-94)
Males/Females	134 (58%)/96 (42%)
Hemoglobin, g/dL, median (range)	13 (6.8-17.2)
Platelets, µL, median (range)	174,736 (15,800-355,000)
WBC, µL, median (range)	29,447 (2970-283,280)
Lymphocytes, µL, median (range)	22,910 (588-268,400)
LDH, mU/ml, median (range)	327 (60-1371)
β2-microglobulin, mg/L, median (range)	3.43 (1- 14.6)
Rai Stage	
0	73 (33%)
1	39 (18%)
2	48 (22%)
3	20 (9%)
4	40 (18%)
Binet Stage	
A	115 (53%)
B	60 (28%)
C	43 (20%)
*TP53* mutation/del(*17p*)	
Present/Wild type	7 (3%)/70 (30%)
Not available	156 (67%)
*IGHV* status	
Mutated/Unmutated	40 (17%)/37 (16%)
Not available	156 (67%)
Comorbidities	
Hypertension	101 (44%)
Ischemic cardiomyopathy	25 (11%)
Dyslipidemia	35 (15%)
Hyper homocysteine	5 (2%)
Diabetes	29 (13%)
Atrial fibrillation	20 (9%)
COPD	24 (10%)
Benign thyroid disease	18 (8%)
GERD	10 (4%)
HCV/HBV-related Hepatopathy	10 (4%)
Stroke/TIA	9 (4%)
BPH	25 (11%)

WBC, white blood cells; IGHV, Immunoglobulin heavy-chain variable region gene; COPD, chronic obstructive pulmonary disease; GERD, gastroesophageal reflux disease; TIA, transient ischemic attack; BPH, benign prostatic hypertrophy.

### 2.2 Endpoints

Efficacy of various anti-CLL therapies was assessed by investigating: time-to-treatment initiation (TTI), defined as the time between diagnosis and treatment start in months; TTNT, calculated as the time between treatment start and subsequent therapy start or patient’s death; TTNF, assessed as the time between the end of a therapeutic treatment and the end of a subsequent therapy or patient’s death; PFS; OS.

### 2.3 Flow cytometry

Flow cytometry immunophenotyping was performed on heparinized peripheral blood (PB) specimens, stained within 12 h from collection using a whole blood lysis technique and directly conjugated antibodies. Briefly, antibodies were added to 200 μL of whole blood, incubated for 20 min at 4°C, lysed with 3 mL of red blood cell lysis buffer, and incubated for additional 15 min at room temperature. Next, samples were centrifuged, and cell pellets were resuspended in 500 μL of phosphate buffer saline (PBS; Gibco, Waltham, MA USA) for acquisition. Antibodies used were: CD45; CD4; CD8; CD3; CD56; CD19; CD5; CD23; CD10; CD11c; CD20; CD103; CD38; CD49d; SmIg-Kappa; and SmIg (all from Beckman Coulter). Sample acquisition was performed on a 10-color three-laser Beckman Coulter Navios Flow Cytometer (Beckman Coulter). Post-acquisition analysis was performed using Kaluza Analysis Flow Cytometry software v2.1.1 (Beckman Coulter). Instrument daily quality control was carried out using Calibrite Beads or Flow-Check Pro Fluorospheres (Beckman Coulter). The Laboratory is enrolled in the international external quality control program UK NEQAS for Leucocyte Immunophenotyping. Samples were run using the same PMT voltages, and at least 500,000 events were recorded.

### 2.4 Statistical analysis

Data were collected in a spreadsheet and analyzed using Prism (v.8.3.0; GraphPad Software, San Diego, CA). Dichotomous variables were compared using Fisher’s exact test, while continuous variables were compared using the non-parametric Mann-Whitney test. Comparisons between two groups were performed using an unpaired t-test. Differences in OS, PFS, TTI, TTNT, and TTNF were evaluated using Log-rank (Mantel-Cox) test, and Hazard Ratio (HR) was calculated by log-rank test. Multivariate analysis was performed using a logistic regression model with SPSS Statistics software (IBM, Armonk, NY). A P-value of <0.05 was considered statistically significant.

## 3 Results

### 3.1 Clinical characteristics at diagnosis

In this retrospective real-life study, a total of 230 consecutive CLL patients were included (median age, 70 years; range 39–94 years; males, 134, 58%; females, 96, 45%). Anemia was present at diagnosis in 41% of subjects (N = 94), thrombocytopenia in 37% (N = 85), leukocytosis in 71% (N = 163), and anemia plus thrombocytopenia in 20% of patients (N = 46). LDH levels were assessed in 123 patients and were increased in 63 cases (51%); β2-microglobulin levels were available in 110 subjects and were found elevated in 53 of them (48%) (**Table 1**). Patients were stratified according to Rai staging systems, and 33% of evaluable subjects (N = 73) were in stage 0, 18% (N = 39) in stage 1, 22% (N = 48) in stage 2, 9% (N = 20) in stage 3, and 18% (N = 40) in stage 4. Based on Binet staging system, 115 were in stage A (53%), 60 in stage B (28%), and 43 in stage C (20%). Most evaluable subjects had *TP53* wild type (wt), while 9% (N = 7) had del(*17p*) or mutated *TP53*. Unmutated *IGHV* was observed in 48% of evaluable patients (N = 37), and 4 of them (5.2%) had also mutated *TP53*; conversely, 40 subjects had mutated *IGHV*, and 3 of them (4%) displayed mutated *TP53*. The most frequent comorbidity was hypertension (44%; N = 101), followed by dyslipidemia (15%; N = 35), diabetes (13%; N = 29), ischemic cardiomyopathy (11%; N =25), benign prostatic hyperplasia (11%; N = 25), chronic obstructive pulmonary disease (COPD; 10%; N = 24), and atrial fibrillation (9%; N = 20).

On the entire cohort, 112 patients (49%) received periodic follow-up visits without pharmacological interventions, while 118 patients (51%) were treated with a first-line therapy, including standard chemotherapy (65%; N = 77) or targeted therapies (35%; N = 41) (**Table 2**). As first-line treatments, most frequently administered standard chemoimmunotherapies were chlorambucil (N = 26, 34%), rituximab + bendamustine (R-BENDA; N = 25, 33%), and fludarabine-cyclophosphamide-rituximab (FCR; N = 9, 12%), while ibrutinib (N = 23, 56%), venetoclax plus obinutuzumab (N = 7, 17%), and acalabrutinib (N = 6, 15%) were the most commonly prescribed targeted therapies. Of those receiving a first-line therapy, 37 of them (31%) underwent a second-line treatment, including standard chemoimmunotherapies in 63% of subjects (N = 22), especially R-BENDA (N = 8, 36.4%) or rituximab alone (N = 5, 23%), and targeted therapies in 13 patients (37%), predominantly with ibrutinib (N = 9, 69%), or venetoclax-based therapies (N = 4, 31%). In details, a second-line therapy was started because of disease progression or intolerance in 33 and 4 patients, respectively. Third- or above lines of treatments mainly included an anti-CLL specific agent, such as ibrutinib, idelalisib, or venetoclax (**Table 2**).

**Table 2:** Therapeutic regimens.

Characteristics	N = 230 (%)
Observation only	112 (49)
First line	118 (51)
Standard chemotherapy	77 (65)
Bendamustine	1 (1)
Bendamustine-rituximab	25 (33)
Chlorambucil	26 (34)
Fludarabine	4 (6)
Fludarabine-alemtuzumab	1 (1)
FC/FCR/FClo	12 (14)
Fludarabine-rituximab	3 (4)
Rituximab only	3 (4)
R-CHOP	1 (1)
R-CVP	1 (1)
Targeted therapy	41 (35)
Acalabrutinib	6 (15)
Ibrutinib	23 (56)
Venetoclax - obinutuzumab	7 (17)
Venetoclax - rituximab	5 (12)
Second line	37 (16)
Standard chemotherapy	22 (60)
Bendamustine-rituximab	8 (36)
Cyclophosphamide	3 (14)
Chlorambucil	3 (14)
Fludarabine	1 (4)
Fludarabine-rituximab	1 (4)
Fludarabine plus R -CVP	1 (4)
Rituximab	5 (23)
Targeted therapy	13 (35)
Ibrutinib	9 (69)
Venetoclax	1 (8)
Venetoclax - rituximab	3 (23)
ASCT	1 (3)
Other	1 (3)
Third line	13 (6)
Standard chemotherapy	5 (39)
Bendamustine - rituximab	2 (40)
Chlorambucil	2 (40)
Fludarabine	1 (20)
Targeted therapy	8 (61)
Ibrutinib	6 (75)
Idelalisib	1 (13)
Venetoclax	1 (13)
Fourth line	4 (2)
Ibrutinib	2 (50)
Rituximab	1 (25)
Venetoclax - rituximab	1 (25)
Fifth line	1
Venetoclax - rituximab	1 (100)

FC, fludarabine- cyclophosphamide; FCR, fludarabine- cyclophosphamide -rituximab; FClo, Fludarabine- Chlorambucil; R-CHOP, Rituximab-cyclophosphamide-doxorubicin-vincristine; R-CVP, Rituximab- cyclophosphamide -vincristine-prednisone; ASCT, autologous stem cell transplantation.

### 3.2 Clinical differences in CLL patients based on first-line therapy choice

To investigate the presence of clinical features that could have influenced first-line therapy choice, patients were divided into three groups based on first-line therapeutic approach: chemotherapy (N = 77); targeted (N = 41); and observation (N = 109) (**Table 3**). Clinical characteristics between groups were first investigated without differences in demographics and comorbidities distribution, except for COPD, that was prevalently represented in patients undergoing standard chemotherapy (N = 14; 18%) (p = 0.019). As expected, most patients with late-stage diseases were in standard chemotherapy or targeted therapy group (p <0.0001) (**Table 3**). No differences in *TP53* mutation or del(*17p*) distribution were observed among groups (p = 0.1752), while mutated *IGHV* was more represented in targeted therapy group (N = 14, 34%) and observational cohort (N = 25; 22%) (p = 0.0002). Mean hemoglobin levels (p = 0.0084) and platelet count (p<0.0001) were higher in patients in the observation arm, while lymphocytosis was lower (p=0.0003). LDH levels were the highest in patients receiving standard chemotherapy (mean, 433.4 U/L) compared to those observed in patients receiving targeted therapy (mean, 298 U/L) and those under observation (mean, 326.7 U/L) (p = 0.0015). Conversely, β2-microglobulin levels were similar between groups (p <0.0001) (**Table 3**). Among cytogenetic abnormalities, del(*13q14*) tended to be more frequently observed in the targeted therapy cohort (N = 16; 39%) (p=0.0683), as well as del(*11q*) (N = 5; 12%) (p = 0.0038), del(*17p13*), and trisomy 12 (20% and 17%, respectively; p = 0.0987 and p = 0.4052) (**Table 4**).

**Table 3:** Clinical features distribution between groups.

Characteristics	Chemotherapy N = 77	Targeted N = 41	Observation N = 112	P value
Median age, years (range)	71	70	69,6	0.2634
Males, n (%)/Females, n (%)	46 (60)/31 (40)	28 (68)/13 (32)	60 (55)/49 (45)	0.2690
Rai stage, n (%)				
0	17 (24)	2 (5)	60 (54)	<0.0001
1	9 (13)	2 (5)	28 (25)	
2	18 (25)	12 (29)	17 (15)	
3	8 (11)	9 (22)	3 (3)	
4	20 (28)	16 (39)	4 (4)	
Binet stage, n (%)				
A	28 (39)	8 (20)	83 (74)	<0.0001
B	25 (35)	13 (32)	24 (21)	
C	19 (26)	19 (46)	5 (5)	
Hemoglobin, median, g/dL, (range)	12,7	12,7	13	0.0084
Platelets, µL, median (range)	153.257	156.350	174.735	<0.0001
WBC, µL, median (range)	50.067	37.914	29.447	0.0170
Lymphocytes, µL, median (range)	39.188	36.291	22.910	0.0003
LDH, mU/ml, median (range)	433,4	298	326,7	0.0015
β2-microglobulin, mg/L, median (range)	4,39	3,9	3,5	<0.0001
Comorbidities, n (%)				
Hypertension, n (%)	35 (45)	13 (32)	44 (39)	0.3577
Ischemic cardiomyopathy, n (%)	7 (9)	10 (24)	11 (10)	0.0503
Dyslipidemia, n (%)	8 (10)	6 (15)	21 (19)	0.2632
Hyper homocysteine, n (%)	2 (3)	2 (5)	1 (1)	0.2631
Diabetes, n (%)	11 (14)	4 (10)	14 (13)	0.8058
Atrial fibrillation, n (%)	8 (10)	3 (7)	6 (5)	0.5005
COPD, n (%)	14 (18)	3 (7)	6 (5)	0.0190
Benign thyroid disease, n (%)	2 (3)	5 (12)	11 (10)	0.0730
GERD, n (%)	2 (3)	5 (12)	5 (5)	0.0862
HCV/HBV-related Hepatopathy, n (%)	5 (7)	2 (5)	3 (3)	0.4302
Stroke/TIA, n (%)	5 (7)	2 (5)	6 (5)	0.9312
BPH, n (%)	5 (7)	3 (7)	17 (15)	0.1347
Allergy, n (%)	4 (5)	2 (5)	1 (1)	0.1546

WBC, white blood cells; IGHV, Immunoglobulin heavy-chain variable region gene; COPD, chronic obstructive pulmonary disease; GERD, gastroesophageal reflux disease; TIA, transient ischemic attack; BPH, benign prostatic hypertrophy.

**Table 4:** Cytogenetics and immunophenotypic alterations distribution between groups.

Characteristics	Entire cohort N = 230	Observation N = 112	Chemotherapy N = 77	Targeted N = 41	P value
Genetic abnormalities					
*TP53* mutation/del(*17p*), n (%)					0.1752
Present/Wild type	7 (3)/69 (30)	1 (1)/32 (29)	3(4)/9 (12)	3 (7)/28 (68)	
Not available	153 (67)	78 (70)	65 (84)	10 (24)	
*IGHV* status, n (%)					0.0002
Mutated/Unmutated	40(17)/37(16)	25(22)/9 (8)	1 (1)/11 (14)	14 (34)/17 (42)	
Not available	153 (67)	78 (70)	65 (84)	10 (24)	
del(*13q14*)	57 (25)	23 (21)	18 (23)	16 (39)	0.0683
del(*11q*)	12 (5)	1 (1)	6 (8)	5 (12)	0.0038
del(*17p13*)	24 (10)	11 (10)	5 (7)	8 (20)	0.0987
Trisomy 12	28 (12)	11 (10)	10 (13)	7 (17)	0.4057
Flow cytometry immunophenotyping					
CD38 positivity					0.0282
CD38+	42 (18)	13 (12)	18 (23)	11 (27)	
CD38-	155 (67)	83 (74)	45 (58)	27 (66)	
Not available	33 (14)	17 (15)	13 (17)	3 (7)	
CD49d positivity					0.4572
CD49d+	53 (23)	24 (21)	18 (23)	11 (27)	
CD49d-	143 (62)	77 (69)	37 (48)	29 (71)	
Not available	34 (15)	11 (10)	22 (29)	1 (2)	
CD11c positivity					0.4779
CD11c+	66 (29)	34 (30)	22 (29)	10 (24)	
CD11c-	137 (60)	69 (62)	38 (49)	30 (73)	
Not available	27 (12)	10 (9)	16 (21)	1 (2)	
CD5 positivity					0.4344
CD5+	198 (86)	99 (88)	61 (79)	38 (92)	
CD5-	15 (7)	9 (8)	3 (4)	3 (7)	
Not available	17 (8)	4 (4)	13 (17)	–	
CD20 positivity					0.4558
CD20+	201 (87)	102 (91)	61 (79)	38 (92)	
CD20-	9 (4)	3 (3)	2 (3)	3 (7)	
Not available	21 (9)	7 (6)	14 (18)	–	
CD38/CD49d co-expression					0.1705
CD38+CD49d-	20 (9)	7 (6)	7 (9)	6 (15)	
CD38+CD49d+	21 (9)	6 (5)	10 (13)	5 (12)	
CD38-CD49d+	27(12)	14 (13)	7 (9)	6(15)	
CD38-CD49d-	109 (47)	60 (53)	29 (38)	20 (49)	

Among immunophenotypic alterations, CD38 positivity was frequent in patients receiving standard chemotherapy (N = 18; 23%) or targeted therapy (N = 11; 27%) (p=0.0282). No significant differences between groups were observed in positivity rates of CD49d (p=0.4572), CD11c (p=0.4779), or CD5 and CD20 (p = 0.4344 and p = 0.4558). Co-expression of CD38 and CD49d was observed in 10 (13%) and 5 (12%) patients treated with standard and targeted therapy, respectively, and only in 6 (5%) subjects under observation. Conversely, double negativity for CD38 and CD49d was mainly documented in patients under observation (N = 60; 53%).

### 3.3 Clinical outcomes and prognostic impact of CD38 expression

Clinical outcomes were compared between CLL patients who were treated with standard chemotherapy as first-line treatment and those who received targeted therapy as a first-line approach (**Table 5**). Patients treated with standard chemotherapy achieved complete response (CR) and partial response (PR) in 70% of cases, while response rate was lower (30%) in targeted therapy group (p < 0.0001) (**Table 5**). In particular, 4 (6%) CR, 2 (3%) PR and 1 (1%) SD for standard chemotherapy arm and 1 (2%) CR, 4 (11%) PR and 6 (16%) SD for targeted therapy group were observed when del(*17p*) or TP53 mutation were detected.

**Table 5:** Comparison of hematological responses based on type and line of therapy.

	Chemotherapy	Targeted	P value
First line	N = 70	N = 37	<0.0001
CR + PR, n (%)	49 (70)	11 (30)	
SD, n (%)	16 (23)	25 (68)	
PD, n (%)	5 (7)	1 (2)	
Second line	N = 19	N= 12	0.2553
CR + PR, n (%)	14 (74)	6 (50)	
SD, n (%)	2 (10)	6 (50)	
PD, n (%)	3 (16)	–	
Third line	N = 5	N = 8	0.2308
CR + PR, n (%)	5 (100)	5 (63)	
SD, n (%)	–	–	–
PD, n (%)	–	3 (37)	

CR, complete response; PR, partial response; SD, stable disease; PD, progressive disease.

Following second-line therapy, response rates tended to be slightly higher in those subjects treated with standard chemotherapy (74%) compared to those who received targeted treatments (50%) (p=0.2553), as well as following third line approach (100% *vs* 63%, standard chemotherapy *vs* targeted therapy; p=0.2308).

**Figure 1:**
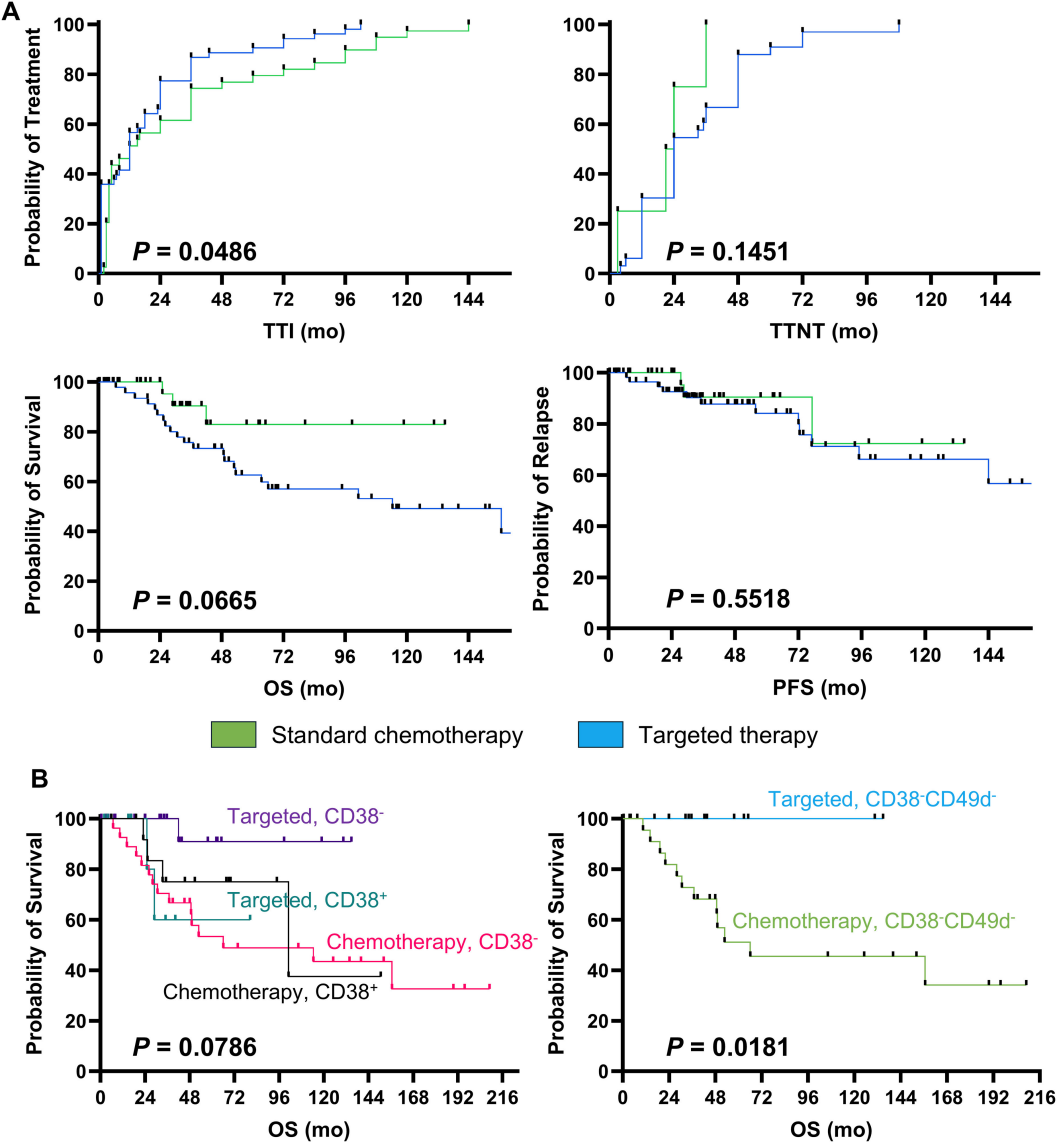
Clinical outcomes. Patients were divided based on type of first-line therapy received, standard chemotherapy and targeted therapy groups were identified, and **(A)** time to treatment initiation (TTI), Time to Next Treatment (TTNT), overall survival (OS), and progression-free survival (PFS) were compared between groups, and Kaplan-Meier estimator curves were used for showing survival analysis. **(B)** Next, patients were stratified based on type of first-line therapy received and positivity or negativity for CD38 and/or CD49d expression on leukemic cells, and OS were compared between groups.

Median TTI was 18 *vs* 33 months in chemotherapy *vs* targeted therapy group, respectively (p = 0.0486), and TTNT was 33 *vs* 21 months (p=0.1451) (**Figure 1A**). In details, FCR was administered earlier (median TTI, 10 months) and those patients had a longer TTNT (52.5 months), while patients treated with R-BENDA regimen started lately this therapy (median TTI, 20 months). Conversely, TTI was longer in the cohort receiving targeted therapy, especially in those subjects treated with anti-CD20 agents + venetoclax regimens (median TTI, 45 months). Outcomes of subsequent therapy lines are reported in **Table 6**. Overall, patients who received targeted therapy as first-line treatment displayed a slightly longer OS compared to those who received standard chemotherapy (p = 0.0665), while no significant differences were documented in PFS (p = 0.5518) (**Figure 1A**).

**Table 6:** Clinical outcomes in refractory/relapsed CLL patients treated with standard chemotherapy or targeted therapy.

	TTI Median, months (range)	TTNT Median, months (range)	P value
Standard chemotherapy	18 (1-102)	33 (4-108)	0.002
R-Benda/Bendamustine	20 (1–102)	30 (4-72)	0.006
Chlorambucil	12 (1-72)	21 (12–36)	0.21
FCR	10 (1-48)	52,5 (12-108)	0.03
Targeted therapy	33 (2–144)	21 (3–36)	0.43
BTKi	27 (2–108)	21 (3–36)	0.43
Anti-CD20 + Venetoclax	45 (3–144)	N.E.	–
	TTNT Median, months (range)	TTNF Median, months (range)	P value
Second line therapies	38 (5-180)	25 (1-84)	0.41
Standard chemotherapy	36 (4-108)	26.3 (1-77)	0.38
R-Benda	62.4	60	0.6
Cyclophosphamide	12 (5-17)	1 (1-17)	–
Chlorambucil	–	16 (1-20)	–
Rituximab	12 (4-18)	22 (1-21)	0.30
Targeted therapy	–	16.9 (1–60)	–
BTKi	–	20.8 (1-60)	–
Anti-CD20 + Venetoclax	–	10.8 (1-12)	–
Third line therapies	15 (12 -21)	21 (1-96)	0.54
Standard chemotherapy	–	–	–
Targeted therapy	15 (6-27)	26.3 (1-96)	0.23

FCR, fludarabine- cyclophosphamide-rituximab; R-Benda, rituximab + bendamustine; BTKi, Bruton’s tyrosine kinase inhibitors.

Prognostic impact of several clinical and phenotypic markers on outcomes was investigated based on type of first-line approach by multivariate analysis (**Table 7**). In details, relapse/refractoriness in the entire CLL cohort was associated with age at diagnosis (p = 0.0041), grade of lymphocytosis (p = 0.003), and platelet count (p = 0.0073), while negativity for CD38 and CD49d markers had a protective effect on PFS (p = 0.0373). However, only patients with negativity for CD38 and treated with targeted therapies showed the highest benefit, as CD38^-^ subjects treated with standard chemotherapy displayed the shortest OS (median OS, not-reached *vs* 66.03 months, CD38^-^ with targeted therapy *vs* CD38^-^ with standard chemotherapy; p = 0.0108). Moreover, co-expression of CD38 and CD49d was significantly related to shorter PFS in patients receiving BTKi or BCL2i, while targeted therapies showed an impressive protective effect in CD38^-^CD49d^-^ CLL/SLL patients compared to standard chemotherapy (5-year OS, 100% *vs* 46%; p=0.0181; **Figure 1B**). Indeed, when multivariate analysis was performed only on patients treated with targeted therapy, patients with a CD38^+^CD49d^+^ phenotype showed a worse outcome (odds ratio [OR], 25.37; 95% confidential interval [CI], 1.56-1219.26; p = 0.0466). Conversely, in the standard chemotherapy group, only age was associated with worse outcomes (OR, 1.09; 95%CI, 1.02-1.18; p = 0.0268) (**Table 7**).

**Table 7:** Outcomes based on first line approach by multivariant analysis.

Entire cohort Dependent variable = relapse/refractory CLL	OR	SE	95% CI	P value
Age	1.05	0.018	1.02-1.09	0.0041
Lymphocyte absolute count	1	0.000007	1.000008 - 1.000036	0.003
Platelet count	0.99	0.000002	0.99 - 1.01	0.0073
CD38^-^CD49d^-^ phenotype	0.47	0.17	0.23 - 0.95	0.0373
CD38^+^CD49d^+^ phenotype	0.29	0.19	0.07 - 0.95	0.0542
Targeted therapy cohort Dependent variable = relapse/refractory CLL	OR	SE	95% CI	P value
Age	0.98	0.046	0.90 - 1.08	0.7401
Lymphocyte absolute count	0.999	0.00007	0.99971 - 0.99998	0.1098
Platelet count	0.999	0.000007	0.99997 - 1.00001	0.1817
CD38^-^CD49d^-^ phenotype	3.14	3.56	0.37 - 35.13	0.31
CD38^+^CD49d^+^ phenotype	25.37	41.19	1.56 - 1219.26	0.0466
Standard chemotherapy cohort Dependent variable = relapse/refractory CLL	OR	SE	95% CI	P value
Age	1.09	0.041	1.02 - 1.18	0.0268
Lymphocyte absolute count	1.000	0.00001	0.99998 - 1.00002	0.9662
Platelet count	0.999	0.000005	0.99998 - 1.00000	0.0706
CD38^-^CD49d^-^ phenotype	0.50	0.47	0.07 - 3.06	0.456
CD38^+^CD49d^+^ phenotype	3.03	2.23	0.74 - 13.87	0.1316

CLL, chronic lymphocytic leukemia; OR, odds ratio; SE, standard error; CI, confidential interval.

**Figure 2:**
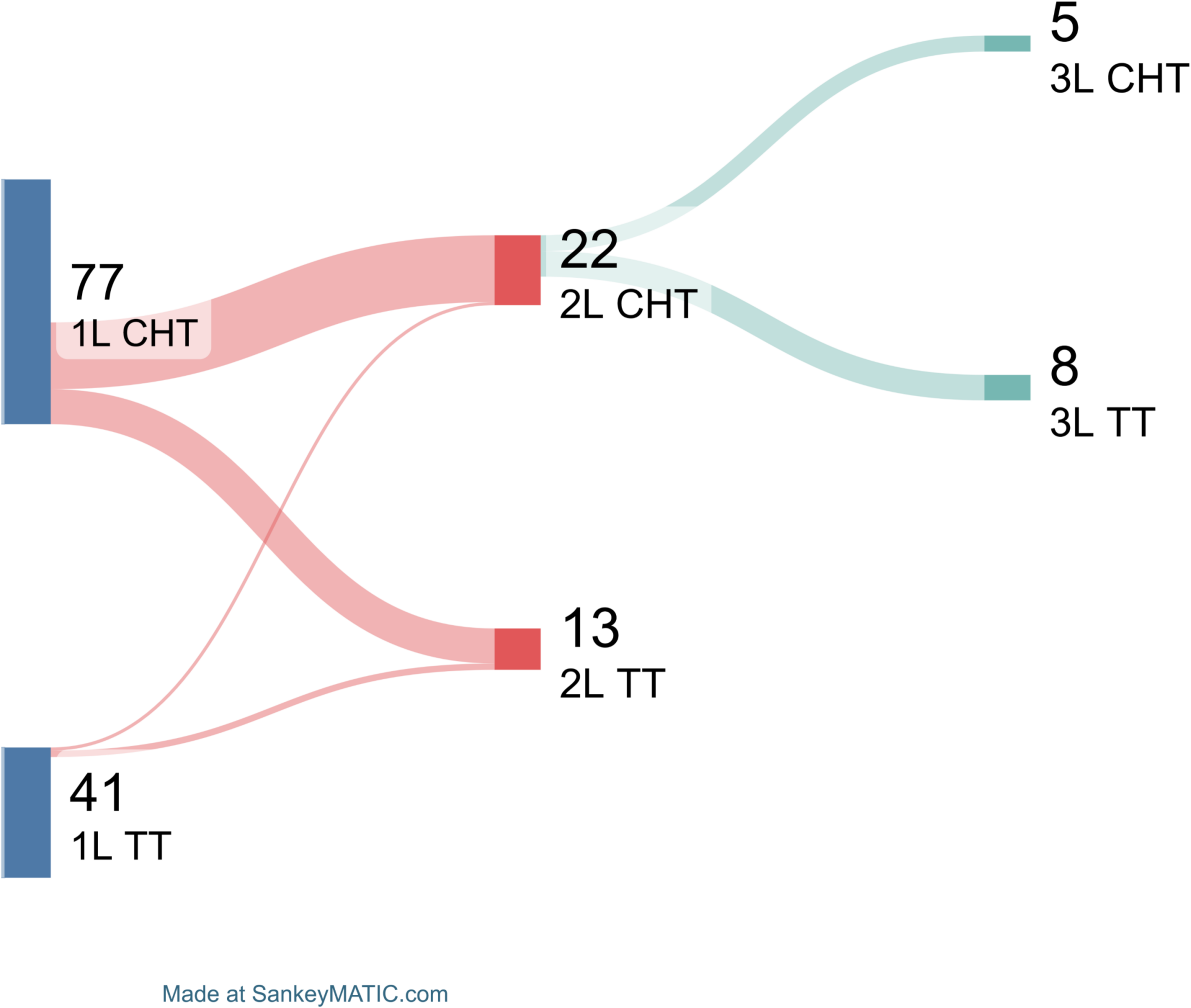
Subsequent treatment line choices. Patients are divided based on first line (1L) treatment type in chemotherapy (CHT) and targeted therapy (TT). Patients treated with second line (2L) therapies are shown, as well as those who received third-line (3L) treatments.

Finally, treatment sequencing was also shown by Sankey plot (**Figure 2**). In details, of total 77 patients treated with chemotherapy as first-line, 21 of them (27%) received chemotherapy also as second line treatment, while 11 (14%) targeted therapy. Of note, among the 21 subjects treated with chemotherapy as second line, 5 of them (24%) received chemotherapy as third line treatment, and 8 (38%) targeted therapy. Conversely, of those treated with targeted therapy as first line strategy, only three subjects received second line therapy (N = 2, targeted therapy; and N = 1, chemotherapy).

## 4 Discussion

CLL is the most common hematologic malignancy in adults in Western countries and is characterized by expansion and progressive accumulation of clonal B lymphocytes in peripheral blood, bone marrow, lymph nodes, and spleen. CLL has high clinical and biological heterogeneity, ranging from indolent forms managed only with an observational approach to more aggressive diseases requiring pharmacological treatments (10). Chemotherapy has been the milestone for CLL treatment for decades, while therapeutic options have significantly expanded in the last decade, with the introduction of continuous therapy with BTK inhibitors and fixed-duration regimens with anti-CD20 monoclonal antibodies (rituximab, obinutuzumab) plus venetoclax, or the recent combination with ibrutinib-venetoclax. Other targeted agents, such as PI3K inhibitors (idelalisib), are less frequently used due to their unfavorable toxicity profile (4, 9). To date, the most appropriate treatment is chosen based on multiple factors, including age, comorbidities, fitness, and cytogenetic/molecular features (e.g., *TP53* abnormalities and *IGHV* mutational status), that highly impact on PFS and OS, as well as disease stage at diagnosis, serological markers related to disease aggressiveness (LDH and β2-microglobulin levels), and immunophenotype (e.g., CD38 and CD49d expression) (11). In this monocentric retrospective study, we described therapeutic CLL strategies and changes over a ten-year period in a real-life context, to add evidence for improving current algorithms for treatment-naïve and relapsed/refractory CLL patients based on biological, phenotypic, and molecular parameters.

Disease stage at diagnosis is a strong independent negative prognostic factor of treatment response; however, there is no clear evidence of clinical superiority of standard chemotherapy versus targeted therapy. For example, in CLL7 trial, patients in Binet stage A have shown no OS benefits when early treated with six FCR cycles (12), while in CLL12, low-risk patients have displayed a better event-free survival when treated with ibrutinib (13). In our study, disease stage at diagnosis significantly influenced clinical outcomes and clinician’s decision to initiate therapy. Patients with intermediate-high Rai (II-III-IV) or advanced Binet stages (B and C) were more likely to early receive a first-line treatment with longer OS, and subjects treated with standard chemotherapy more frequently achieved a hematological response, compared to those who started a targeted therapy.

Other well-known prognostic markers are hematologic (hemoglobin, platelets, and lymphocytes) and serological (LDH and β2-microglobulin) parameters, that are currently included in prognostic scores, also guiding therapeutic approach and strategy (14, 15).

Here, we confirmed that severe lymphocytosis associated with low platelet count was a risk factor for disease progression and treatment refractoriness, while the presence of CD38^+^CD49d^+^ phenotype was a risk factor of disease progression or non-responsiveness to targeted therapies used as first-line approach. Conversely, advanced age was a prognostic factor in patients receiving standard chemotherapy. Indeed, in elderly, Binet stage, ZAP70, β2-microglobulin levels, and comorbidity can stratify patients, by identifying a group of CLL subjects with shorter OS, such as those with multiple comorbidities at treatment start (16).

Cytogenetic abnormalities in CLL are various and contribute to clinical heterogeneity of this disease (17). Patients carrying del(*17p*) or del(*11q*) have a more aggressive disease compared to those with del(*13q*), as well as those subjects with unmutated *IGHV*, somatic mutations in *TP53*, *ATM*, or *NOTCH1*, or increased expression of certain microRNAs (18). In our cohort, cytogenetic abnormalities tended to be more frequent in those subjects receiving targeted therapies as first-line approach, as well as *TP53* abnormalities and unmutated *IGHV* status. Moreover, we confirmed that CLL patients with mutated *IGHV* and del(*13q*) had a less aggressive disease, prevalently treated with an observational or targeted therapy approach.

High levels of ZAP70 and CD38 are associated with reduced survival in CLL patients, especially in those with unmutated *IGHV*, and CD38 positivity is frequently found in combination with anemia, thrombocytopenia, and leukocytosis (19). However, no definitive results on clinical utility of CD38 are present, as well as for CD49d. This alpha4 integrin (also known as CD49d) is frequently expressed on resting CLL cells and is a negative independent prognostic factor associated with short OS and TTI, alone or in association with CD38 and/or CD11c expression (20). Indeed, CD49d interacts with CD38 and forms a macromolecular complex involved in several biological mechanisms leading to increased survival and migration abilities of CLL cells (21). CD49d negativity is the most common condition in CLL patients, often associated with CD38 and/or CD11c negativity and low serum β2-microglobulin levels. Conversely, CD49d co-expresses with CD38, CD11c, and unmutated *IGHV* (22, 23). CD49d^+^ CLL patients are more likely to receive therapy with shorter TTI, especially those subjects with CD49d^+^CD38^+^ phenotype (24). In this ten-year observational study, we confirmed that absence of CD38 and CD49d expression was related to better clinical out-comes, principally in patients receiving targeted therapies (N=49), while not in those under standard chemotherapy approach (N=38). Indeed, CD38 highly influences cell metabolism and surface expression of VCAM-1/CD106 on stromal/endothelial cells, leading to CLL cell survival and anti-apoptotic signals upon CD38 binding (25). Moreover, CD38 co-localizes with CD19 and CD81 on CLL cell surface, causing a B-cell receptor signaling and amplification and enhancing proliferative and survival effects. Blocking of CD38 downstream results in decreased p-Syk, p-BTK, p-ERK1/2 and p-AKT: these two latter are even more reduced in CD38^-^ patients, and simultaneous inhibition of CD38 and BTK in CLL cell from CD38- patients leads to a significant downregulation of only p-BTK, p-PLCγ2 and p-ERK1/2 (26). Therefore, our real-life results suggested that targeted therapies could be more effective than standard chemotherapy in both CD38^+^ and CD38^-^ CLL patients, because of more selective BTKi and BCL2i pro-apoptotic effects on downstream signaling in neoplastic cells.

In fit patients without *TP53* mutations or del(*17p*) and with mutated IGHV, fixed-duration therapy with FCR remains an effective treatment option despite growing use of targeted therapy (7). According to literature, our patients with aggressive and advanced-stage CLL benefited more from first-line standard chemotherapy in terms of TTNT compared to targeted therapy. In the phase III FLAIR study, efficacy and safety of ibrutinib-rituximab has been compared to FCR, without significant differences in OS, despite a PFS advantage (27). Similarly, long-term results from the phase III A041202 study, no positive impact on OS have been described for ibrutinib-treated *vs* bendamustine-rituximab-treated older CLL patients, while only a PFS advantage (28), supporting the use standard chemotherapy as first-line approach in fit patients without significant comorbidities. In contrast, in the RESONATE-2 study, efficacy of ibrutinib monotherapy versus chlorambucil has been investigated, showing longer OS and PFS in patients receiving targeted therapy (29). Therefore, for CLL patients without predictive biological characteristics of high disease aggressiveness, targeted therapy is recommended, as confirmed in our real-life study. Our CLL patients on targeted therapy as first-line approach had longer 5-year OS compared to those on standard chemotherapy, especially in elderly patients with non-aggressive disease and comorbidities. Recently, ibrutinib + venetoclax have been approved in clinical practice, following phase II CAPTIVATE study (NCT02910583) results (30), and this novel combination regimen may change the future therapeutic landscape towards an increasingly widespread use of fixed targeted therapies. Indeed, standard chemotherapy has been progressively replaced by targeted therapies, as they are safer and more manageable with lower incidence of toxicities, including cytopenias, infections, and secondary malignancies, and higher efficacy compared to chemotherapy, especially in those subjects with *TP53* mutations, del(*11q*), and IGHV unmutated (31–34).

Choosing the best therapeutic approach for relapsed/refractory CLL patients is complex, because several factors need to be considered, such as age, gender, comorbidities, type of first-line therapy, patient preferences (including therapy duration), disease characteristics, risk profile, and the depth of response to first-line approach (35). Moreover, biologic features, including mutational IGHV status, and immunophenotypic characteristics should be also considered (36). Despite the small cohort of patients receiving second- or subsequent lines of treatments, our study indicated therapeutic lines based on targeted regimens positively impacting patient survival, especially in those subjects with molecular and phenotypic alterations.

Strengths of our retrospective study are: (i) monocentric nature of data collection, ensuring uniformity in patient diagnosis and treatment criteria, homogeneity in clinical, biological, phenotypic, and molecular data in accordance with the specific period; and (ii) accurate record of therapy start and stop dates. Limitations of our study are: (i) limited availability of long-term data for patients on first-line targeted regimens and lack of molecular biology findings in several patients due to diagnosis made outside our Center or before introduction of these markers in routinely clinical practice, or impracticality to perform these tests; (ii) retrospective nature of this study; (iii) long collection period resulting in great variability in available anti-tumor agents; (iv) absence of certain prognostic markers in patients diagnosed in the first decade of 2000; (v) relatively small cohort size due to the monocentric nature of this investigation; (vi) absence of geriatric evaluation; and (vii) poor representation of more innovative agents, such as acalabrutinib, zanubrutinib, or ibrutinib-venetoclax, given the historical nature of our cohort.

## 5 Conclusions

In conclusion, treatment selection in CLL patients must involve a preliminary assessment of clinical (disease stage, age, fitness), biological (*IGHV* and *TP53* status, hematologic and serological parameters), and molecular factors (7). Standard chemotherapy and even more targeted regimens are effective in clinical trials, as well as in a real-life setting, as confirmed in our study, in particular in young CLL patients with advanced-stage disease and markers of aggressiveness. Moreover, elderly with advanced-stage disease in the absence of negative prognosticators, such as positivity for CD38 and CD49d (N=5 and N=6 for age 66–75 and ≥75 years, respectively), might benefit more from a targeted therapy approach. However, observation periods must be extended to better assess the impact of standard chemotherapy and targeted therapy on clinical outcomes. Indeed, given the recent introduction of novel combinations of targeted agents into clinical practice, clinical management and outcomes of CLL patients might further change. Therefore, continuous enrolment of CLL patients in new perspective real-life clinical trials is mandatory to better define treatment sequencing and strategies in CLL based on a personalized approach including clinical, biological, phenotypic, and molecular features.

## Data Availability

The original contributions presented in the study are included in the article/supplementary material. Further inquiries can be directed to the corresponding author.
